# Genetic Alchemy unveiled: MicroRNA-mediated gene therapy as the Artisan craft in the battlefront against hepatocellular carcinoma—a comprehensive chronicle of strategies and innovations

**DOI:** 10.3389/fgene.2024.1356972

**Published:** 2024-06-10

**Authors:** Abduh Murshed, Mohammed A. H. Alnoud, Saleem Ahmad, Safir Ullah Khan, Mohammed Alissa, Meshari A. Alsuwat, Ahmed Ezzat Ahmed, Munir Ullah Khan

**Affiliations:** ^1^ Department of Intensive Care Unit, Affiliated Hospital of Guangdong Medical University, Zhanjiang, China; ^2^ Cardiovascular Center of Excellence, Louisiana State University Health Sciences Center, New Orleans, LA, United States; ^3^ Hefei National Laboratory for Physical Sciences at the Microscale, School of Life Sciences, University of Science and Technology of China, Hefei, China; ^4^ Department of Medical Laboratory, College of Applied Medical Sciences, Prince Sattam bin Abdulaziz University, Al-Kharj, Saudi Arabia; ^5^ Department of Clinical Laboratory Sciences, College of Applied Medical Sciences, Taif University, Taif, Saudi Arabia; ^6^ Department of Biology, College of Science, King Khalid University, Abha, Saudi Arabia; ^7^ Prince Sultan Bin Abdelaziz for Environmental Research and Natural Resources Sustainability Center, King Khalid University, Abha, Saudi Arabia; ^8^ MOE Key Laboratory of Macromolecular Synthesis and Functionalization, International Research Center for XPolymers, Department of Polymer Science and Engineering, Zhejiang University, Hangzhou, China

**Keywords:** hepatocellular carcinoma, gene therapy, microRNA, therapeutics 1.Introduction, liver cancer

## Abstract

Investigating therapeutic miRNAs is a rewarding endeavour for pharmaceutical companies. Since its discovery in 1993, our understanding of miRNA biology has advanced significantly. Numerous studies have emphasised the disruption of miRNA expression in various diseases, making them appealing candidates for innovative therapeutic approaches. Hepatocellular carcinoma (HCC) is a significant malignancy that poses a severe threat to human health, accounting for approximately 70%–85% of all malignant tumours. Currently, the efficacy of several HCC therapies is limited. Alterations in various biomacromolecules during HCC progression and their underlying mechanisms provide a basis for the investigation of novel and effective therapeutic approaches. MicroRNAs, also known as miRNAs, have been identified in the last 20 years and significantly impact gene expression and protein translation. This atypical expression pattern is strongly associated with the onset and progression of various malignancies. Gene therapy, a novel form of biological therapy, is a prominent research area. Therefore, miRNAs have been used in the investigation of tumour gene therapy. This review examines the mechanisms of action of miRNAs, explores the correlation between miRNAs and HCC, and investigates the use of miRNAs in HCC gene therapy.

## 1 Introduction

Hepatocellular carcinoma (HCC), often referred to as liver cancer, poses a significant global health challenge (Aly et al., 2020). It is the fourth leading cause of cancer-related fatalities and the sixth most widespread illness worldwide. Approximately 80% of all liver cancer cases are attributed to HCC, which is the primary cause of cancer-related deaths (Bray et al., 2018). Despite advances in medical treatments, late-stage HCC remains difficult to treat, with patients typically having a life expectancy of only 6 months after diagnosis (Jemal et al., 2010). A range of factors, such as age, cirrhosis, genetic predisposition, toxin exposure, excessive alcohol consumption, and smoking contribute to the likelihood of developing HCC. Furthermore, individuals infected with hepatitis B or C viruses are at an increased risk. Recently, there has been a surge in the recognition of NAFLD and its severe form, non-alcoholic steatohepatitis (NASH), as critical precursors of HCC in Western countries. Notably, NAFLD and NASH have emerged as primary causes of liver transplantation after HCV infection (Welzel et al., 2013; Bellentani, 2020). The management of HCC depends on the stage of the disease. Radiofrequency ablation (RFA) is the preferred treatment for small HCC tumours (≤2 cm) because it is similar in effectiveness to surgical excision, causes minimal tissue damage, and allows for faster recovery. However, the negative impact of hypoxia-induced vascular development restricts the effectiveness of transcatheter arterial chemoembolisation, which is typically used for intermediate-stage HCC (Llovet et al., 2008). Although there has been a slight increase in patient survival, sorafenib, a multi-target kinase inhibitor, is currently the most effective systemic therapy for advanced unresectable HCC with limited therapeutic alternatives (Kane et al., 2009; Zhang et al., 2019).

The use of natural substances such as hemobasine has shown promise in controlling the production of microRNA-16 (miR-16), which could be beneficial for treating hepatocellular carcinoma (HCC) (MacFarlane and Murphy, 2010). However, further investigation is necessary to confirm these findings. MicroRNAs (miRNAs) are non-coding RNA molecules that bind to specific messenger RNA (mRNA) targets in order to regulate gene expression (Yao et al., 2019). They play a role in various biological processes, including signalling pathways, cellular proliferation, and programmed cell death. Specific miRNAs, such as miR-122, miR-199, miR-221, and miR-21, have been linked to the progression of hepatocellular carcinoma (HCC) (Gramantieri et al., 2008). Among these, miR-199a and miR-122 are the most abundant miRNAs in normal liver tissues. Disruption of these molecular pathways occurs throughout the development of HCC, and these miRNAs have the potential to influence multiple therapeutic targets, including P53, RAS/MAPK, WNT/β-catenin, PI3K/AKT/mTOR, MET, MYC, and TGF-β (Hou et al., 2011).

**FIGURE 1:**
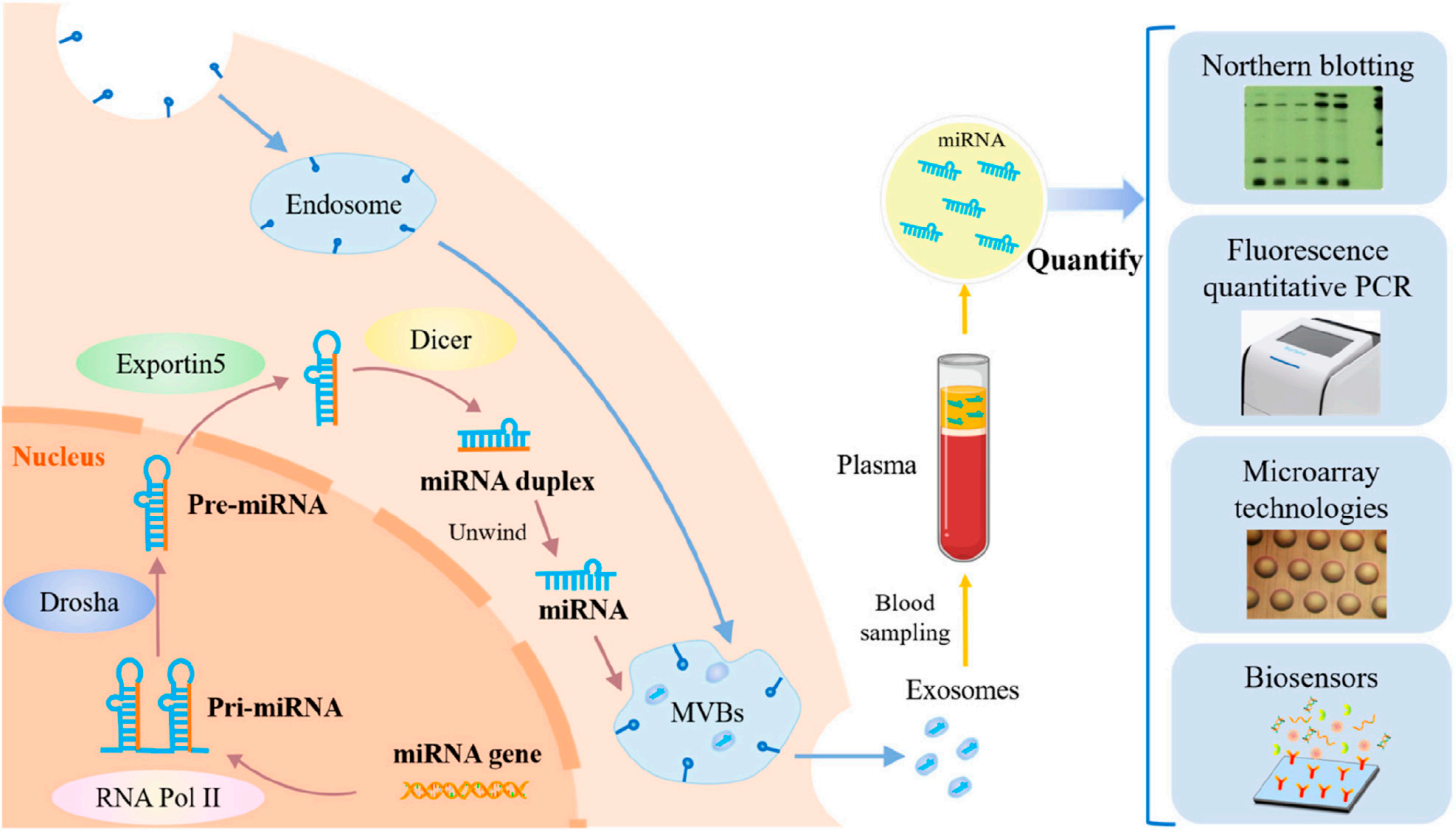
An overview of cancer cell miRNA synthesis, miRNA extraction, and miRNA quantification techniques. (Reproduced from Lin X et al., 2023, licensed under CC BY 4.0)

Researchers have demonstrated by examining liver cancer genomes that non-coding RNAs (ncRNAs), including miRNAs, lncRNAs, and circRNAs, play a critical role in disease development (Ghidini and Braconi, 2015). These transcripts affect gene transcription, stability, and translation (Rowe and Kaestner, 2023), but cannot code for proteins themselves. The development of hepatocellular carcinoma (HCC) is associated with an imbalance in miRNA expression, which interacts with important regulators of cell cycle pathways and directly influences the proliferation of HCC cells (Kamel et al., 2016). Therefore, miRNAs show great potential as therapeutic targets and indicators of HCC. On average, microRNAs (miRNAs) are 22 nucleotides long and have a wide range of functions in cancer cell regulation (Song et al., 2021). The commonly used quantitative approaches for miRNAs in cancer cells are depicted in Figure 1, along with the steps involved in their synthesis, acquisition, and application.

The development of HCC is accompanied by abnormal regulation of several miRNAs. However, miRNAs are challenging to identify in complex biological materials because of their short sequences, low *in vivo* quantity, and susceptibility to degradation (Chen et al., 2018). Traditional detection techniques, including qRT-PCR, microarray, and Northern blotting, have several limitations. Although qRT-PCR and microarrays offer many advantages, such as sensitivity and dynamic range, contamination remains possible. Quick screening is possible using microarray technology; however, this requires sophisticated probes, equipment, and trained professionals. Northern blotting has a reputation as the “gold standard” method, but it has drawbacks such as long detection durations, substantial sample consumption, and low sensitivity (Zhang L. et al., 2022).

Consequently, researchers are currently investigating novel and efficient therapies. Gene therapy, a form of biological cancer treatment, has gained increasing attention owing to rapid advancements in molecular biology and gene engineering technologies. Gene therapy involves the introduction of therapeutic genes into cells affected by diseases to rectify the genes responsible for the disease or to compensate for faulty genes to achieve therapeutic outcomes (Shen et al., 2013; Yuan et al., 2013). The primary investigations in gene therapy for combating tumours encompass the following: incorporation of tumour suppressor genes, suppression of proto-oncogenes, integration of suicide genes, introduction of genes that impede tumour growth, and stimulation of the self-anti-tumour immune response (Chen et al., 2013). Gene therapy offers distinct benefits, such as precise targeting and high specificity, and holds significant potential for further research (Lemay et al., 2012; Chang et al., 2013). They significantly affect the control of biological processes, including the creation of organs, differentiation, apoptosis, and cell proliferation (Alvarez-Garcia and Miska, 2005). Several diseases, including cancer, are closely related to miRNA dysregulation, which may cause changes in the cell cycle, unchecked growth, and programmed cell death (Iorio and Croce, 2012). This review article examines the mechanism by which miRNAs operate, studies the relationship between miRNAs and hepatocellular carcinoma, and considers the potential use of miRNAs in gene therapy for HCC. Furthermore, we investigated the manipulation of miRNAs for potential therapeutic and diagnostic applications in HCC.

## 2 Biogenesis of canonical MicroRNAs: a quick overview

MicroRNAs are critical in regulating genomic expression, impacting various cellular functions, including metabolism, signaling pathways, autophagy, hematopoiesis, and programmed cell death. New data indicate that miRNAs and microbiota may be significantly correlated (Rani and Sengar, 2022). The process of miRNA formation commences with the transcription of particular genes that encode miRNAs, which RNA polymerase II regulates in the nucleus. The outcome of this process is the creation of an miRNA (Lee et al., 2004). Then, the ribonuclease complex DiGeorge Syndrome Critical Region 8 (DGCR8) Drosha (ribonuclease III) cleaves the pri-miRNA, producing precursor miRNA (pre-miRNA), which has a sequence of up to 100 nucleotides and a shorter length of ≥1000 nucleotides (Lee and Shin, 2018; O'Brien et al., 2018).

Another step involves the transport of pre-miRNAs into the cytoplasm by exportin 5 and the RAS-related nuclear protein guanosine-50-triphosphate (Ran-GTP). An additional mechanism by which the RNA-binding protein TRBP and RNase III endonuclease Dicer cleave cytoplasmic pre-miRNAs exists (Shi et al., 2019). Dicer eliminates the terminal loop of the pre-miRNA sequence. Once the terminal loop is removed, miRNAs undergo a process called duplex formation, during which they briefly form a double-stranded structure and integrate into an RNA-induced silencing complex (RISC). The RISC complex, consisting of Argonaute RISC Catalytic Component 2 (Ago2), separates the mature miRNA strand (leading strand) from the passenger strand, causing the latter to decay (Doench and Sharp, 2004). Furthermore, the mRNA’s 3′-untranslated region (3′UTR) is targeted explicitly by miRNA-RISC (miRISC), which finds and binds to an mRNA strand that is only partly complementary. Crucial to this process is the miRNA seed sequence, which profoundly affects the interaction of miRNAs with mRNA (Jungers and Djuranovic, 2022; Koustas et al., 2023).

**FIGURE 2:**
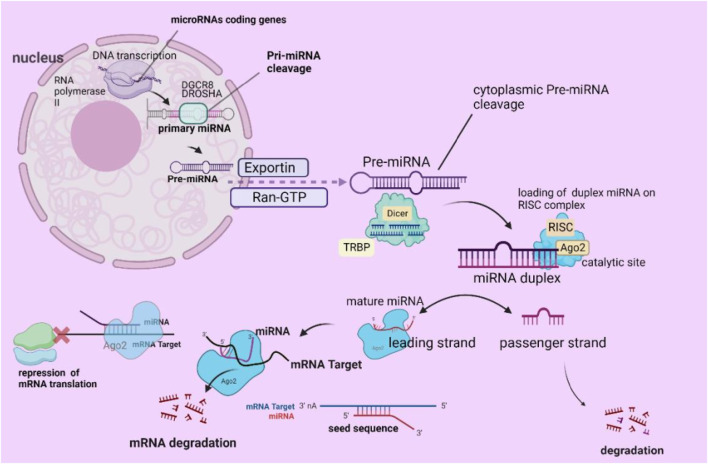
Diagrammatic depiction of the process of miRNA biogenesis. (Reproduced from Koustas et al., 2023, licensed under CC BY 4.0)

The miRNA strand binds to the partially complementary mRNA sequence, inhibiting mRNA translation and ultimately suppressing or breaking mRNA (Yousef et al., 2022). Notably, another biogenesis pathway functions separately from the Drosha-DGCR8 and DICER complexes (Naeli et al., 2023), an illustrative depiction of standard miRNA formation (Figure 2).

## 3 Mechanism of action of miRNA

Noncoding short RNAs (miRNAs) comprise an average of approximately 22 nucleotides. The nucleases Dicer and Argonaute interact with the TAR RNA-binding protein TRBP and double-stranded RNA-binding protein PACT to produce an RNA-induced silencing complex (RISC) (PKR) (Bhaskaran et al., 2019). By forming complementary base pairs, the RISC complex aids in the binding process between the 3′UTR of the target mRNA and the 6-to 8-base seed sequence located at the 5′end of the miRNA. Degradation of the target mRNA occurs if the two are complementary. Partial complementary pairing prevents translation of target mRNA. Therefore, a single miRNA can target numerous mRNAs simultaneously; conversely, many miRNAs can target a single mRNA (Shukla et al., 2011). In addition to blocking translation via their primary method, miRNAs may bind to the mRNA’s coding sequence and 5′untranslated region (UTR). Promoter sequences can bind to and initiate the transcription of specific target genes. Protein translation is triggered by binding to the AU-rich region of the target mRNA (Kunej et al., 2012). Hence, miRNAs can exert regulatory control over mRNA and protein levels, thereby governing cellular processes such as growth, differentiation, and apoptosis.

## 4 Relationship between miRNA and HCC

One of the main areas of attention in tumour research is the exploration of the connection between miRNAs and tumour initiation and progression. Genomic advancements have paved the way for high-throughput technologies, such as gene chips and real-time quantitative PCR. Owing to these technological advancements, differences in miRNA expression may be quickly detected in tumour tissues compared to normal tissues and even within tumour tissues. To further our understanding of miRNA target genes, they also facilitate the creation of tumor miRNA expression profiles (Huang et al., 2009; Wong et al., 2010). This study thoroughly investigated the levels of miRNA expression in both HCC and non-cancerous tissues around HCC in individuals diagnosed with HCC. Many miRNAs show aberrant expression patterns unique to HCC tissues (Murakami et al., 2006; Wang et al., 2008). Hepatocellular carcinoma (HCC) is a major liver disease that often begins with viral hepatitis. According to previous research, liver cancers caused by different viruses have different microRNA (miRNA) expression patterns. Other viruses are also associated with the expression of various microRNAs (miRNAs). In contrast, different miRNAs are associated with different stages of illness development through their expression (Ura et al., 2009).

Moreover, an unhealthy lifestyle, such as drinking, is linked to the unusual expression of miRNAs (Ladeiro et al., 2008). Researchers have compared miRNA expression profiles in liver cancer tissues from two groups of patients with HCC with distinct prognoses (Jiang et al., 2008). They found a strong association between the expression levels of the 19 miRNAs and prognosis. Therefore, miRNAs can serve as predictive biomarkers. Studies investigating the same miRNA, such as miR-122, have shown varying aberrant expression statuses (Connolly et al., 2008; Varnholt et al., 2008). These differences may be explained by parameter variations, such as the source of the samples, stage of disease development, and detection methods used. Therefore, more research is necessary to authenticate the current discoveries.

Based on their characteristics, miRNAs can be classified into two groups: cancer-inhibiting and cancer-causing. Tumour formation and progression are typically associated with decreased levels of tumour-suppressing miRNAs and increased expression of cancer-causing miRNAs. miRNAs exert their inhibitory effects on proto-oncogenes by explicitly targeting their mRNA, leading to the degradation or suppression of translation. This ultimately hinders the carcinogenic effects of these genes. Reducing the expression of miRNA results in the activation of proto-oncogenes, which in turn promotes the development of tumours. Carcinogenic miRNAs exhibit opposite effects. Suppressing the entire process of miRNA maturation enhances the likelihood of tumour development, thereby increasing the anti-cancer effects of miRNAs (Kumar et al., 2007). MiR-122, a liver tissue-specific miRNA, is the most commonly researched miRNA for its anti-cancer properties. Its expression is reduced in liver cancer tissues.

It was discovered that miR-122 could specifically bind to the anti-apoptotic gene Bcl-w, reducing both its mRNA and protein levels (Lin et al., 2008; Shi et al., 2019). This ultimately facilitates apoptosis. The presence of the Cyclin G1 gene is prominent in numerous cancers, and its overexpression can impede the tumour-suppressing function of P53. In 2009, scientists discovered that miR-122 can decrease the expression of Cyclin G1 protein by specifically targeting Cyclin G1 (Fornari et al., 2009). This action removes the inhibitory impact of Cyclin G1 on P53, suppressing tumour growth. The Wnt/β-catenin-TCF signalling pathway is crucial for the initiation and progression of liver cancer. MiR-122 can specifically bind to Wnt1, decreasing the amounts of Wnt1, β-catenin, and TCF-4 proteins. This, in turn, leads to the inhibition of tumour cell development and the promotion of tumour cell death through a process known as apoptosis.

The extensively researched oncogenic miRNA miR-221 exhibits increased expression in 70% of HCC tissues accompanied by cirrhosis. miR-221 can control the cell cycle by reducing the cell cycle suppressor proteins CDKN1C/p57 and CDKN1B/p27 (Fornari et al., 2009; Xu et al., 2012). Bcl-2 modifying factor (BMF) is a protein that promotes programmed cell death (apoptosis) and is involved in anoikis. It is crucial to balance the signalling pathways that promote cell death with those that prevent it. Another study showed that miR-221 can suppress the expression of Bmf, preventing programmed cell death in nonadherent cells (Gramantieri et al., 2009). Tissue inhibitors of metalloproteinases (TIMPs) may impede the action of matrix metalloproteinases (MMP), hindering cell invasion and metastasis. Research conducted in 2009 showed that miR-221 and miR-222 can suppress the expression of PTEN and TIMP3 in HCC tissues (Garofalo et al., 2009). It facilitates the development of tolerance to TNF-related apoptosis-inducing ligand (TRAIL), an experimental anticancer medication currently undergoing clinical trials, while stimulating cellular proliferation, migration, and invasion.

miRNAs can directly or indirectly control crucial functional genes, including those associated with apoptosis and metastasis, leading to aberrant cellular alterations. It is also feasible to control one or more genes in the signalling route, influencing the operation of the entire signalling system. The complexity of miRNA regulatory networks arises from the diverse mechanisms that underlie miRNA regulation. Variations in miRNA expression are also associated with the resistance of HCC patients to anticancer medications, potentially due to the influence of miRNA-regulated genes and drug target sites. Hence, miRNAs can offer valuable insights into tailored therapies.

## 5 Application of miRNA in the treatment of HCC

The carcinogenic or anticancer properties of miRNAs have generated significant interest among researchers, leading to the exploration of miRNA-based gene therapy. Currently, there are three approaches to managing HCC using miRNAs. One approach is to use miRNA antagonists to suppress the activity of oncogenic miRNAs. miRNA antagonists are nucleic acid compounds that can bind to and complement endogenous miRNAs, thereby inhibiting the processing of endogenous miRNAs by RISC or promoting their destruction of endogenous miRNAs. Examples of miRNA antagonists include anti-miRs, locked nucleic acids (LNAs), and antagomiRs. Currently, researchers are studying miRNA-specific small chemical inhibitors that suppress miRNA activity. The second approach involves augmenting the expression levels of naturally occurring cancer-inhibiting miRNAs and lowering the expression of their target proto-oncogenes. Typical techniques involve the direct administration of synthetic miRNA. Expression vectors containing cancerous DNA or mimics that replicate endogenous cancer-suppressive miRNA sequences in cells introduce miRNA genes into cells to inhibit their activity (Bader et al., 2010). The latter approach is commonly employed because most miRNAs in tumour cells are suppressed (Lu et al., 2005). Furthermore, artificial microRNA (miRNA) expression vectors target genes associated with malignant tumor characteristics (Liu et al., 2012).

When considering gene therapy, it is essential to select vectors that are efficient, safe, and targeted. Viral vectors, such as adenovirus, adeno-associated virus, herpes simplex virus, retrovirus, lentivirus, and poxvirus, are utilised in over 70% of gene therapy research (Vannucci et al., 2013). The notable benefits of viral vectors are their exceptional efficacy in delivering genetic material and their straightforward synthesis and purification processes for *in vivo* studies. Nevertheless, the utilisation of viral vectors in humans is now restricted because of the potential danger of genetic material recombination, which can lead to cancer and trigger immunological reactions in the body (Yoshimura et al., 2010). Owing to the ongoing debate surrounding the safety of viral vectors, achieving the required amount of DNA for clinical applications requires time and effort. Therefore, there has been a push to advance research on non-viral vectors. The most straightforward method is Naked DNA, which can be directly administered into the target area to induce expression (Ginn et al., 2013). Nanoparticles are a recently invented type of carrier, with nanoliposomes as an early example. Nanoparticles have the potential to enhance the effectiveness and capacity of drugs by increasing their ability to be absorbed by the body and carry a more significant amount of medication. They can also reduce the need for frequent and high doses of drugs, target specific tissues or cells, minimize harm to healthy cells, improve the safety and sustainability of drug delivery, overcome barriers in the body’s protective layers, and transport multiple drugs simultaneously (Miele et al., 2012). Transposon methods such as Sleeping Beauty (SB) and piggyBac (PB) are reliable, efficient, and remarkably potent for indirect *in vivo* therapies involving several types of human stem cells (Di Matteo et al., 2012). Multiple gene therapy protocols for HCC have been initiated in human trials, including herpes simplex virus thymidine kinase and a recombinant adenovirus vector containing p53 (Guan et al., 2011). Utilizing the HSV-TK gene generation adenovirus vector in conjunction with conventional therapy can enhance the survival rate of patients with advanced HCC (Sangro et al., 2010; Yang et al., 2010).

Nevertheless, there are no reports on the use of mirNa-based gene therapy for HCC. Aberrant miRNA expression is intricately linked to disease, and mirNa-based gene therapy aims to rectify this aberrant expression. Correcting the aberrant expression of a single miRNA is typically sufficient (Kota et al., 2009).

In 1998, it was discovered that it successfully suppressed the activity of the target gene by employing double-stranded RNA (dsRNA) with a sequence similar to that of the target gene (Fire et al., 1998). This technique is known as RNA interference (RNAi). In 2001, another scientist successfully implemented RNA interference (RNAi) in human cells by utilizing 21-22 nucleotides of small interfering RNA (siRNA) generated through cleavage of double-stranded RNA (Elbashir et al., 2001). RNA interference (RNAi) has emerged as a prominent method for gene therapy. Natural miRNAs operate through an endogenous RNA interference (RNAi) process, and gene therapy that utilises natural miRNAs uses natural endogenous RNAi to suppress the expression of specific genes. Artificial miRNA utilises the precursor structure of natural miRNA (microRNA precursor, pre-miRNA) but substitutes its native core sequence with a sequence that selectively disrupts target gene production. The suppressive effect of artificial microRNAs (miRNAs) on target genes is more potent than that of short hairpin RNA (shRNAs) (Maczuga et al., 2012).

Furthermore, miRNAs are controlled by a polymerase II promoter, enabling tissue-specific or regulated expression (Boudreau et al., 2009). Key considerations include optimal safety, minimal toxicity, absence of disruption of cellular endogenous RNAi, and limited off-target effects (Boden et al., 2004; McBride et al., 2008). Since 2008, multiple laboratory studies on HCC gene therapy utilizing miRNAs have demonstrated specific tumour-inhibitory effects in living organisms and controlled environments, establishing a basis for subsequent clinical trials and developing safe and efficient approaches for patients with HCC.

### 5.1 Natural miRNA-based HCC gene therapy

In a study conducted in 2010, it was discovered that 72 h after introducing miR-29a/b/c mimics into HepG2 cells, the cells exhibited heightened susceptibility to apoptosis triggered by hypoxia, serum starvation, and doxorubicin chemotherapy (Lin et al., 2014). The rate of apoptosis was approximately double that of the control group. Tumour formation rates decreased by 75% on day 10, and tumour volume decreased by 95% after 4 weeks in female BALB/c nude mice injected subcutaneously with miR-29b mimics. In a separate study, researchers used miR-199a/b-3p mimics to transduce four distinct types of liver cancer cells—Hep3B, SMMC-7721, Huh7, and HepG2—that expressed low levels of miR-199a/b-3p (Ren et al., 2016). This method returned miR-199a/b-3p expression levels to normal levels. The number of apoptotic cells increased by approximately 85%–185% while the viability of Hep3B cells decreased by approximately 15%–30% 72 h after transfection. In the G1 phase, the cell population increased by approximately 10%, whereas in the G2 phase, it decreased by approximately 55%. Subsequently, SMMC-LTNM was developed as a nude mouse model of human HCC, derived from human HCC tissues implanted under the skin (Huang M. et al., 2019). They then administered cholesterol-modified miR-199a/b-3p mimics directly into the tumour tissues of the animals. The expression levels of miR-199a/b-3p rose to around 1.4 times those of normal human tissue after 72 h.

• Concurrently, the tumour volume was reduced by approximately 50%, while the serum alpha-fetoprotein (AFP) level was decreased by approximately 45%. Additionally, notable necrosis of the tumour tissue was detected.

The plasmid containing the gene employed a tissue-specific promoter, enabling selective expression of the target gene in specified tissues while excluding others. A vector called AFP-hTERTmiRNA26a (pATM) regulates the production of miR-26a, a molecule that targets estrogen receptor α (ERα) (Lin et al., 2019). This vector was explicitly and effectively designed to express miR-26a in hepatocellular carcinoma cells. Following the injection of PATM-transfected Huh-7 cells under the skin of nude mice, the size of the tumour decreased by approximately 75% over 5 weeks. Twenty micrograms of pATM were combined with liposomes and administered intratumorally to SMMC-LTNM nude mice twice weekly for 2 weeks. As a result, the tumour volume decreased by approximately 60% after 4 weeks. In 2023, a famous scientist developed an miR-214 expression vector called pLL3.7-pre-214. This vector was designed to target the Zeste homologue 2 (EZH2) enhancer, the human equivalent of the Zeste gene found in fruit flies (Jangam et al., 2023). Following the transfection of HLE and SK-HEP-1 hepatoma cells, cell growth was reduced by approximately 30% and 25%, respectively. Furthermore, the number of colonies formed by SK-HEP-1 decreased by approximately 50% after 72 h. Slovakia: Subcutaneous injection of BALB/c nude mice with HEP-1 cells expressing miR-214 resulted in a significant reduction of approximately 70% in tumour volume after 8 weeks.

They used the self-complementary adeno-associated virus scAAV8 to create the adeno-associated virus vector scAAV8.miR-26a. eGFP. This vector specifically targeted the cell cycle components Cyclin D2 and Cyclin E2. The vector was administered to mice by injecting it into their tail veins. The expression of miR-26a was normalised. Out of the ten mice injected with the virus, the average number of liver tumours decreased by approximately 65%. However, the virus did not effectively inhibit tumour growth in two cases, owing to low transduction efficiency. In the remaining eight cases, the virus successfully suppressed tumour growth, even in cases where the tumours were not visually detectable.

In addition, the virus did not cause any visible damage to normal liver cells or other standard tissue cells. This study revealed that the anticancer effect of miR-26a is strongly linked to its ability to suppress tumour cell growth and trigger tumour cell death. lentimiR-125b, a lentiMir-125b vector that produces miR-125b to target the oncogene LIN28B, was developed (Takashima et al., 2016). They then infected HepG2 and Huh7 hepatoma cells with this vector for 4 days. The cell growth rate decreased by approximately 20% and 40% in HepG2 and Huh7 cells, respectively. Nude mice were subcutaneously injected with Huh7 cells expressing consistent levels of miR-125b. After 4 weeks, a significant decrease of approximately 65% in tumour weight was observed. In 2011, Hou administered the adeno-associated virus vector AAV8-miR-199a/b-3p, which contains miR-199a/b-3p that targets the phosphorylase gene PAK4, to SMMC-LTNM nude mice (Hou et al., 2011). The injection was administered through the tail vein twice weekly for 3 weeks. After 4 weeks, there was an observed reduction of almost 50% in tumour volume and a fall of approximately 45% in AFP levels.

## 6 Effects of miRNA on the microenvironment of hepatocellular carcinoma

The tumour microenvironment (TME) of hepatocellular carcinoma (HCC) is a complex area composed of cellular and non-cellular components. Hepatic stellate cells (HSCs), endothelial cells, immune cells, and cancer-associated fibroblasts (CAFs) are important stromal cells in HCC. In the TME, these entities collaborate to produce multiple extracellular components, including the extracellular matrix (ECM), cytokines, growth factors, proteolytic enzymes, and other proteins (Novikova et al., 2017). Insufficient arterial blood flow, rapid oxygen consumption by tumour cells, and specific metabolic alterations all contribute to a hypoxic environment, which is characteristic of HCC (Chen and Lou, 2017). HCC tumours can increase in size, invade surrounding tissues, and spread to other parts of the body owing to their irregular vascular structure and excessive formation of new blood vessels (angiogenesis) (Yao et al., 2023). A comprehensive analysis demonstrated that microRNAs (miRNAs) play a critical role in angiogenesis in HCC. For instance, reduced levels of miR-140, miR-26a, miR-195, and miR-503 and increased production of vascular endothelial growth factor (VEGF) stimulate angiogenesis (Hou et al., 2020). Inhibition of miR-214 leads to a hypervascular increase in HCC by activating the paracrine pathway of hepatocellular carcinoma-derived growth factor (HDGF) (Shih et al., 2012).

**FIGURE 3:**
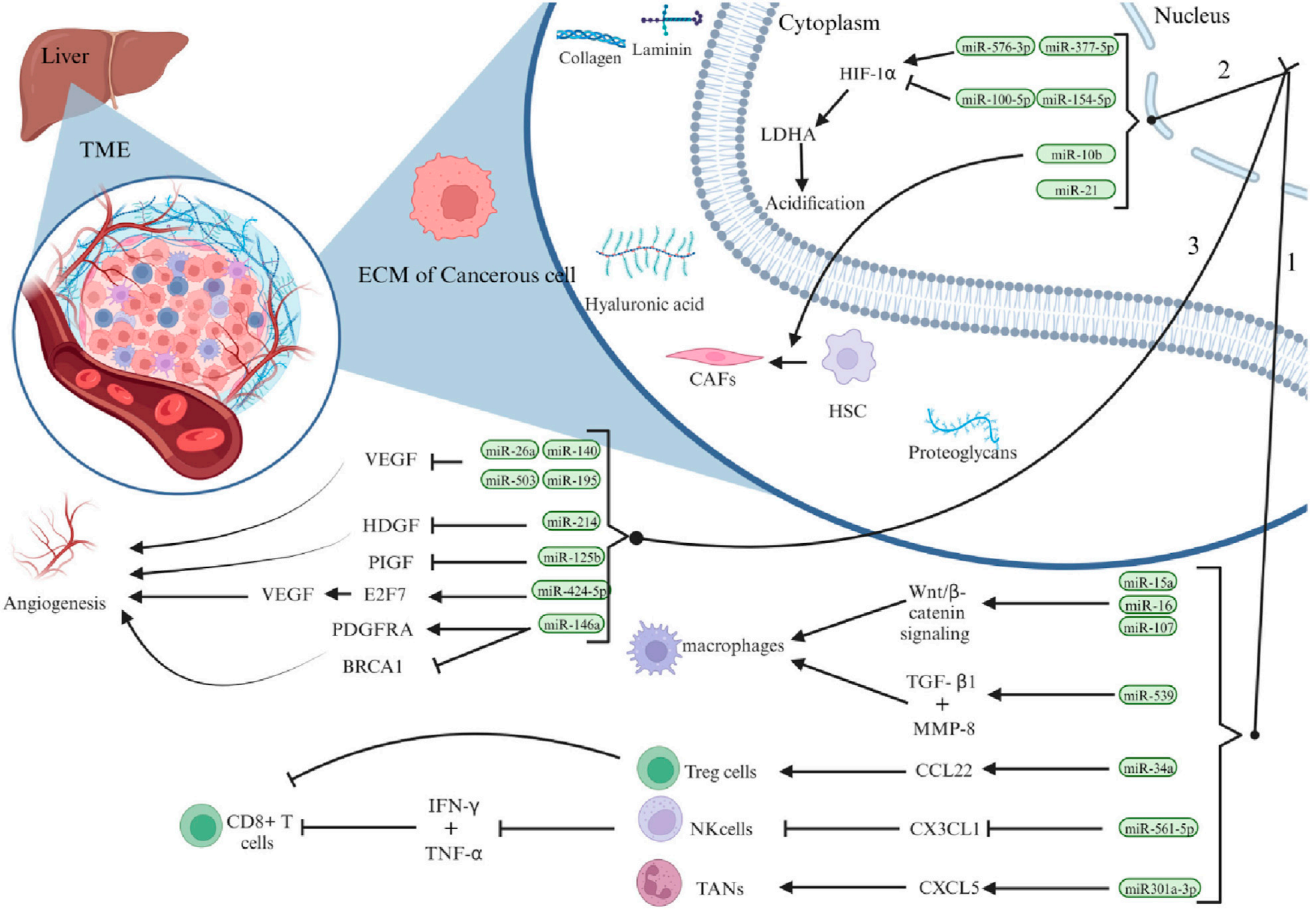
Illustrates the significant role that miRNAs play in shaping the hepatocellular carcinoma (HCC) microenvironment. Specifically, liver cancer cells influence HCC progression through three primary mechanisms: first, by regulating the density of immune cells within the tumour microenvironment (TME); second, by promoting acidification through aerobic glycolysis and hypoxia; and third, by stimulating vascular proliferation. (Reproduced from Tavakoli Pirzaman et al., 2024, licensed under CC BY 4.0)

miRNAs play a crucial role in the regulation of resistance of hepatocellular carcinoma (HCC) cells to chemotherapeutic agents such as paclitaxel. One of the mechanisms by which miRNAs regulate this resistance is by controlling the expression of pro-apoptotic proteins, such as Bcl-2 antagonist killer 1 (Bak1), through the regulation of miR-125b (Apollonova et al., 2023). In contrast, some miRNAs, such as miR-146a, stimulate the growth of new blood vessels in the tumour microenvironment (TME) by regulating the expression of breast cancer 1 (BRCA1) and platelet-derived growth factor receptor alpha (PDGFRA) to enhance angiogenesis in HCC endothelial cells (Zhu et al., 2013). Recent studies have also demonstrated a link between miR-424-5p and increased angiogenesis and cell proliferation in HCC through overexpression of E2F7 and activation of vascular endothelial growth factor receptor 2 (VEGFR-2) signalling (Teng et al., 2020). In addition to regulating angiogenesis, changes in the composition of the extracellular matrix (ECM) in solid HCC tumours have a significant impact on tumour activity, including their ability to migrate and proliferate. Studies have shown that miR-1246 and miR-34a, produced by hepatic stellate cells (hsc) and mesenchymal stem cells (MSCs), stimulate the Wnt/β-catenin signalling pathway, impeding the function of E-cadherin, disrupting the connections between cells, and triggering the transformation of epithelial cells into mesenchymal cells, known as epithelial-mesenchymal transition (EMT) (Huang et al., 2020), as shown in Figure 3.

## 7 Analyzing MicroRNA dysregulation: discovering mechanisms and consequences in hepatocellula carcinoma

MicroRNAs play a crucial role in the complex field of cell regulation, and they are often found in vulnerable and breakpoint-prone genomic regions. This link is strongly associated with cancer development (Calin et al., 2004). In hepatocellular carcinoma (HCC), variability in miRNA expression reflects the presence of genomic instability, which is influenced by several molecular abnormalities that interfere with the precise regulation of miRNA gene expression (Anwar et al., 2013). Various chemical changes, such as mutations and histone modifications, have long-lasting effects on the miRNA landscape, thereby influencing its development (Anwar and Lehmann, 2014). An interesting example of this molecular interaction involves histone deacetylases, which are responsible for maintaining chromatin integrity. In hepatocellular carcinoma (HCC), the activity of these enzymes increases, leading to a chain of events that ultimately results in the inhibition of miR-449. This miRNA is a key regulator of c-Met, a protein that controls cell fate (Buurman et al., 2012). The increase in c-Met activity caused by a reduction in miR-449 expression creates a strong defence against cell death and promotes an environment that supports uncontrolled cell growth. He et al., 2011 further explored this issue, revealing a puzzling increase in miR-191 expression in HCC tumours relative to non-tumour tissues, which is an indicator of prognostic importance. Their study revealed a correlation between hypomethylation events and increased expression of miR-191, which triggers a transition to a mesenchymal phenotype characterized by migration and invasiveness. On the other hand, when the downregulation of miR-191 was reversed, it indicated a shift in the cell state back to a less aggressive state. Although the details of miRNA dysregulation are unknown, it is not necessary to understand them in order to create biomarkers. However, exploring the detailed molecular mechanisms underlying this disorder has the potential to reveal hidden aspects of disease development, providing several potential targets for therapeutic interventions.

## 8 Advancements in future therapeutic strategies

Recent studies have advanced the understanding and utilization of microRNAs (miRNAs) and their potential applications in cutting-edge medicine. Significant advancements have been made by utilizing the complex interactions between miRNAs, autophagy, and the gut microbiome (Zhang H. et al., 2022). Within the context of liver cancer development, miRNAs play a dual function, with their expression levels being precisely regulated to either prevent or enhance tumour growth for efficient tumour suppression (Pratama et al., 2019). One example is enhancing miR-122’s tumor-suppressing action by targeting ADAM10, IGF1R, cdk G1, and ADAM17. Blocking these targets concurrently may improve the expression of miR-122 (Castro-Muñoz et al., 2023). Furthermore, decreased miR-296 levels are linked to poor outcomes in HCC (Shi et al., 2018). Its overexpression via interaction with the fibroblast growth factor receptor 1 (FGFR1) gene slows tumour development, initiates programmed cell death, and controls the cell cycle (Wang et al., 2016).

Furthermore, miR-199, a tumour suppressor that is significantly downregulated in HCC, is a potential therapeutic target (Guo et al., 2015). Specifically, miR-199a-5p plays a complex role in controlling glycometabolism by decreasing the levels of glycolytic products (ATP and G6P). This action restricts the growth of HCC cells by inhibiting glucose uptake in cancerous liver cells (Bozzato et al., 2022). Trans-arterial chemoembolisation (TACE) is a recommended therapeutic technique that exploits the heightened sensitivity of hepatocytes to short RNA sequences to deliver miR-122. Tumour invasion and growth are dramatically reduced when miR-92a activity is inhibited (Wang et al., 2017). However, increased expression of miR-29a hinders the spread of cancer cells to other parts of the body by targeting VEGFA, LOXL2, and LOX (Yang et al., 2021). Furthermore, suppressing oncogenic miR-203a-3p.1, a direct target of IL24, impedes the spread of HCC (Huo et al., 2017).

Additionally, increased miR-940 expression leads to programmed cell death. It inhibits HCC formation, an effect similar to that observed when oestrogen-related receptor gamma (ESRRG), a target of miR-940, is inhibited (Yuan et al., 2015). Utilizing autophagomiRs and other targets in the autophagy pathway presents new opportunities for enhancing HCC management. By inhibiting FBXW7 protein, targeting miR-25 effectively overcomes HCC resistance to sorafenib (Xie and Sun, 2019; Feng et al., 2022). The function of miR-375 was improved to make malignant hepatocytes more responsive to sorafenib by suppressing cytoprotective autophagy (Yang et al., 2020). Furthermore, upregulated levels of miR-559 can inhibit the growth of HCC, drug resistance, and formation of new blood vessels by reducing the expression of PARD3 (Wang et al., 2022). miR-221 and miR-122 mimics, which are examples of miRNA mimics and inhibitors, have been found to have beneficial effects by effectively decreasing cell growth, signs of inflammation, and the formation of new blood vessels [189]. The efficacy of MRX34, an miR-34a mimic, was evaluated in a phase I clinical trial (NCT01829971) involving advanced solid tumours, including HCC (Hassan et al., 2023). However, the end of the study period was driven by significant adverse effects. As a result, lower doses were used in subsequent phase II studies of HCC (Hong et al., 2020). The use of AuNPs as therapeutic agents for miRNAs shows potential for restoring miRNA physiological function and overcoming medication resistance. The use of gold nanoparticles (AuNPs) to deliver an miR-326 mimic is an effective method to suppress the PDK1/AKT/c-myc axis and regulate many processes such as invasion, migration, apoptosis, and epithelial-mesenchymal transition (EMT) in HCC (Mo et al., 2019).

Finally, by promoting autophagy-induced miR-224 degradation, the off-label use of amiodarone, a commonly used antiarrhythmic medication, may function as an HCC suppressor (Huang S.-T. et al., 2019).

## 9 Summary and future perspective

MicroRNAs (miRNAs), a non-coding RNA, regulate gene expression significantly. HCC can arise from a combination of aberrant causes. miRNAs play crucial roles in the onset and progression of HCC. Current research has primarily examined the regulatory function of miRNAs, with little investigation into the mechanism of miRNA alterations in the onset and progression of HCC. Studying the regulatory mechanisms of aberrant miRNA expression will enhance our understanding of the molecular processes underlying the onset and progression of HCC and offer novel therapeutic concepts and approaches. The use of miRNA features in gene therapy has generated significant interest among researchers, with miRNA-based gene therapy emerging as a novel and productive approach for treating HCC. Currently, the application of miRNAs in HCC treatment is still in the experimental phase. Although some laboratory and animal studies have been conducted, no trials have yet been conducted. Simultaneously, gene therapy encounters challenges, including the efficacy of vector administration, the potential for prolonged and stable expression, and safety concerns. Hence, the primary concern is to determine the most effective and secure vectors for miRNA-based treatment of HCC. MV and exosomes serve as inherent mediators for intercellular transmission of diverse signalling molecules and have the potential to be vehicles for delivering therapeutic natural or synthetic miRNAs, representing a promising avenue for future investigation. Owing to advancements in biomedical technology and the accumulation of research expertise, miRNA-based gene therapy for HCC is anticipated to progress to the clinical trial phase and ultimately be employed to treat patients with HCC.
